# Partnerships, Processes, and Outcomes: A Health Equity–Focused Scoping Meta-Review of Community-Engaged Scholarship

**DOI:** 10.1146/annurev-publhealth-040119-094220

**Published:** 2020-01-10

**Authors:** Kasim Ortiz, Jacob Nash, Logan Shea, John Oetzel, Justin Garoutte, Shannon Sanchez-Youngman, Nina Wallerstein

**Affiliations:** 1 Department of Sociology and Criminology, University of New Mexico, Albuquerque, New Mexico 87131, USA; 2 College of Population Health, Center for Participatory Research, University of New Mexico, Albuquerque, New Mexico 87131, USA; 3 Institute for the Study of “Race” and Social Justice, University of New Mexico, Albuquerque, New Mexico 87131, USA; 4 Waikato Management School, University of Waikato, 3240 Hamilton, New Zealand; 5 Behavioral Health Research Center of the Southwest (BHRCS), Pacific Institute for Research and Evaluation (PIRE), Albuquerque, New Mexico 87106, USA; 6 Center for Social Policy, University of New Mexico, Albuquerque, New Mexico 87131, USA; 7 School of Public Administration, University of New Mexico, Albuquerque, New Mexico 87131, USA

**Keywords:** community-based participatory research, CBPR, community-engaged research, CEnR, participatory action research, PAR, participatory health research, community–academic partnerships, CAPs, research-to-practice partnerships, RPPs, CBPR conceptual model

## Abstract

In recent decades, there has been remarkable growth in scholarship examining the usefulness of community-engaged research (CEnR) and community-based participatory research (CBPR) for eliminating health inequities. This article seeks to synthesize the extant literature of systematic reviews, scoping reviews, and other related reviews regarding the context, processes, and research designs and interventions underlying CEnR that optimize its effectiveness. Through a scoping review, we have utilized an empirically derived framework of CBPR to map this literature and identify key findings and priorities for future research. Our study found 100 reviews of CEnR that largely support the CBPR conceptual framework.

## INTRODUCTION

In the last three decades, participatory research has become a well-recognized strategy to improve health equity (16, 17). Several forms of participatory health research resonate for population health sciences, such as community–academic partnerships (CAPs; 54), participatory action research (PAR; 92), youth participatory action research (YPAR; 102), action research, research practice partnerships (RPPs; 37, 71), citizen science (41), and the most well-known being community-based participatory research (CBPR; 129). Since the 2006 inception of the Clinical Translational Science Awards (CTSA), the National Institutes of Health (NIH) has more broadly adopted the terminology of community-engaged research (CEnR) to denote participatory-oriented research (46). CBPR and CEnR arose partially in response to historical research abuse within communities of color and other marginalized communities, wherein inequitable research relationships perpetuated deep-seated mistrust, with data often not returned to the community and community benefit not considered. The NIH has integrated CEnR as key to reducing health inequities across disease conditions, increasing minority enrollment in research, diversifying the health workforce, augmenting implementation science, and enhancing external validity of research findings (40). A 2017 National Academies of Science (NAS) report clarifies the intermediary role that community-driven solutions play in achieving health equity (96), including informing collaborative efforts with local knowledges’ and contexts (66, 76, 88). We have provided definitions to provide greater clarity in differentiating these concepts. See the Supplemental Definitions for further explanation of these terms.

Developing shared understandings of what constitutes CEnR in population health is difficult, however, as the many terms used to describe collaborative research come from different disciplines and epistemic orientations, though some draw from one another. For example, Eder and colleagues’ CEnR logic model, within the CTSA context, draws heavily upon CBPR (58). CBPR has unique origins found within the social justice movements of the Global South [exemplified by the work of Paulo Freire (62, 130) and Arnstein’s ladder of participation (6)]. PAR and RPPs, on the other hand, have origins within educational sciences. Fragmentation of these terms can fuel disparate evaluative trajectories resulting from the varied languages each subfield deploys, possibly diminishing the effectiveness of community-driven solutions. Despite these differences, for the purposes of this review, we utilize CEnR as an umbrella term to describe community-participatory and community-engaged research efforts.

## AN EMPIRICALLY DERIVED CBPR MODEL

Following the groundbreaking seminal review of CBPR published by Viswanathan et al. in 2004 (127), CBPR investigators were challenged to strengthen conceptual models for future research investigations and translational efforts. In 2006, the University of New Mexico (UNM)’s Center for Participatory Research received pilot NIH–National Institute of Minority Health and Health Disparities (NIMHD) funding through its Native American Research Centers for Health (NARCH) mechanism to partner with the University of Washington (UW)’s Indigenous Wellness Research Institute for an exploratory study of CBPR. Through extensive literature reviews of articles and measures, community partner consultations, and guidance from a national advisory committee of academic and community CBPR experts, this pilot produced a CBPR conceptual model with four domains. These domains include research contexts (i.e., environments, policies, funding, historic trust/mistrust), partnering processes (structural and relational dynamics among partners), intervention and research designs as outputs of shared decision making, and broad CBPR and health outcomes. One of the impetuses for the creation of the CBPR model was the realization of the need to legitimize CBPR/CEnR as a science. Constructing an empirically derived model that elucidates the most salient aspects of partnering processes that shape outcomes strategically would facilitate continued federal funding support.

**Figure 1:**
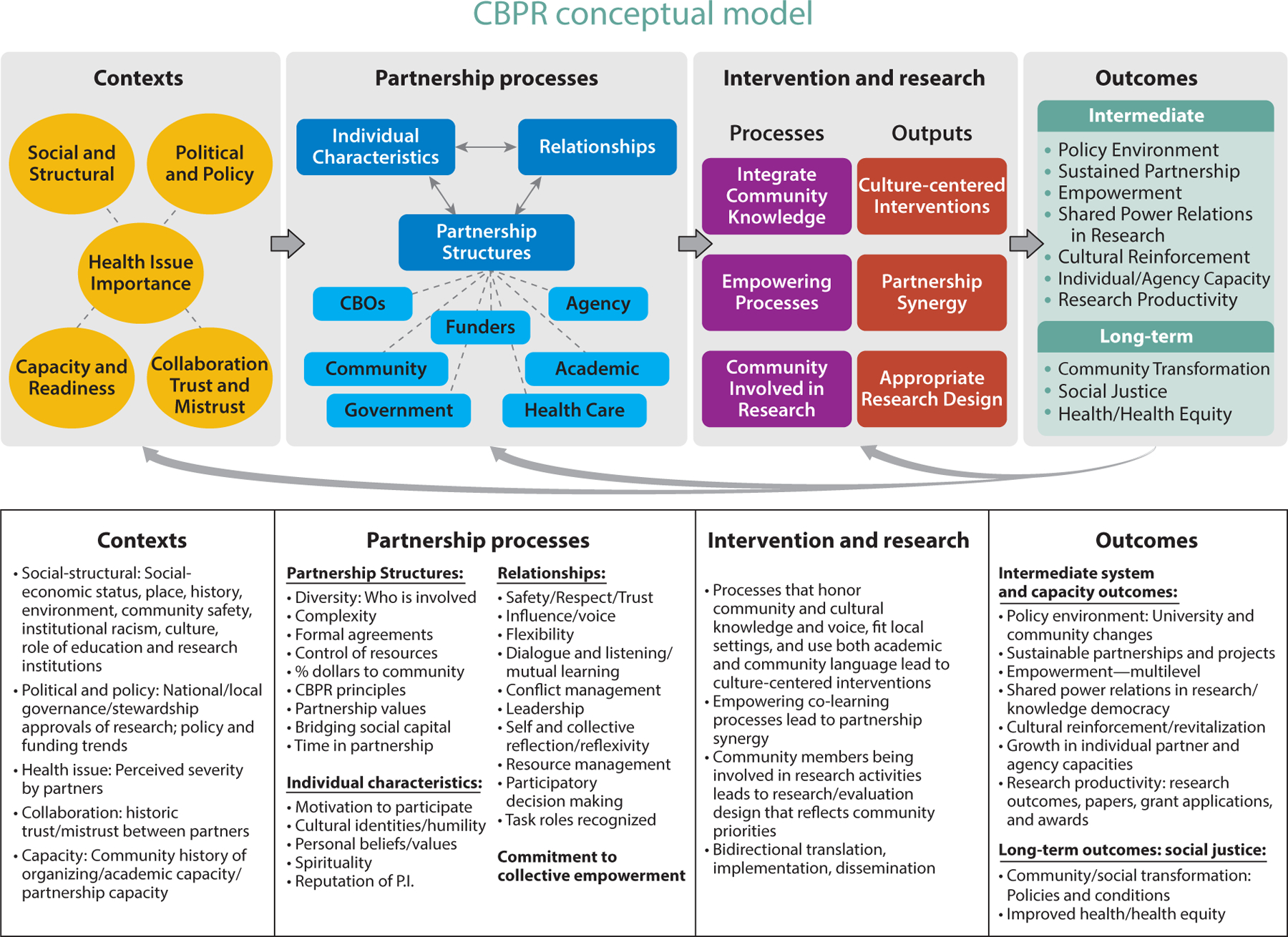
CBPR conceptual model. Abbreviations: CBO, community-based organization; CBPR, community-based participatory research; P.I., principal investigator. Figure adapted with permission from References 129, 133, https://cpr.unm.edu/research-projects/cbpr-project/cbpr-model.html. Visual adapted with permission from Amos Health and Hope, 2017, https://www.amoshealth.org/.

Following the pilot, the UNM–UW collaboration, along with the National Congress of American Indians Policy Research Center, secured the four-year Research for Improved Health (RIH) NARCH grant to test the model and pilot measures of engaged practices and outcomes with Internet surveys of 200 federally funded partnerships across the United States and 7 in-depth case studies (47, 73, 103). Thus, several analyses were undertaken, including validation of the psychometric properties of the subscales comprising the CBPR model (101), evaluation of acceptable concepts across each domain (112), assessment of face validity (11), and initial analyses of associations between partnering and outcomes (34, 57, 100, 132). The current NIH–National Institute for Nursing Research (NINR)-funded Engage for Equity (E^2^) grant seeks to further the science with new surveys collected from a national sample of federally funded partnerships (*n* = 179) and 36 new partnerships (134). These efforts culminated in the current CBPR model (see Figure 1).

**Figure 2:**
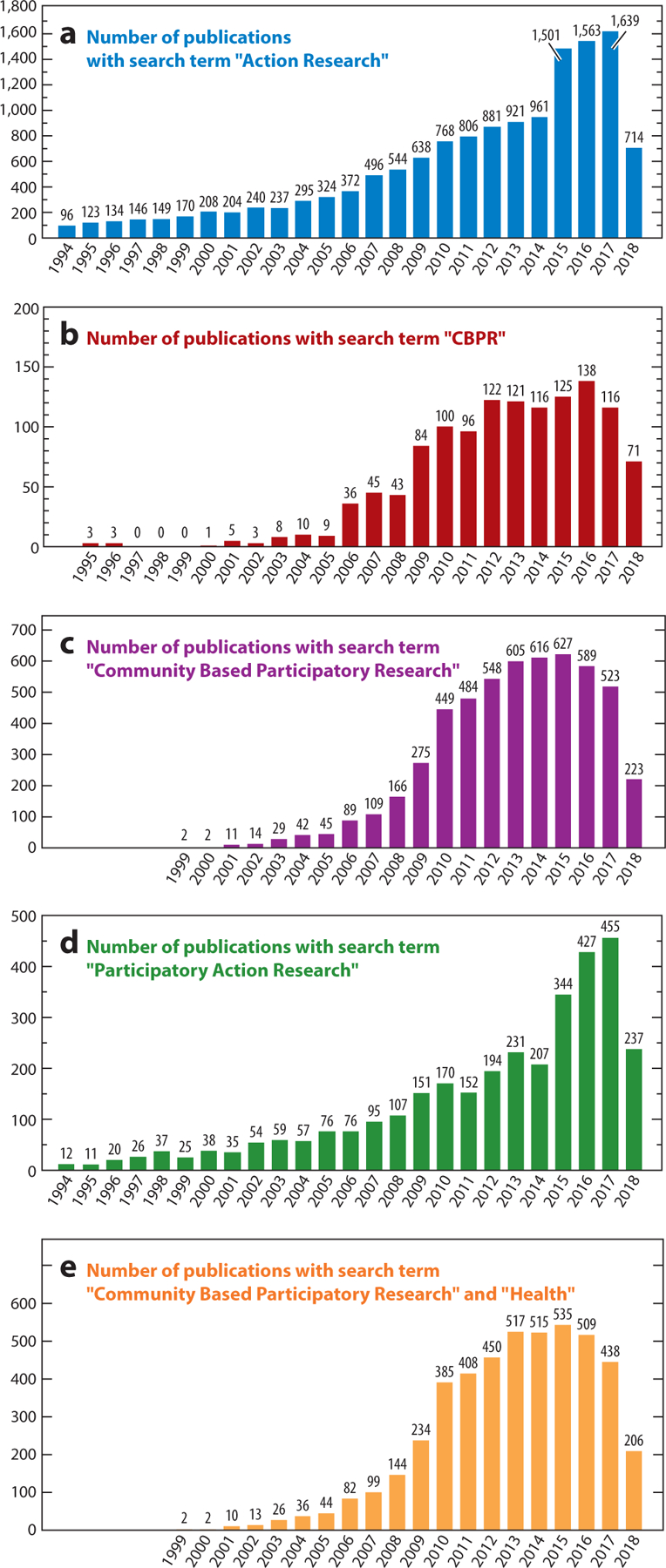
Charts displaying frequency of publications with key search terms.

In this study, we seek to synthesize the extant literature regarding CEnR through a scoping meta-review, using the four domains from the above CBPR model as an analytic structure: contexts; partnering processes, intervention, and research designs; and intermediate and long-term outcomes. To contextualize the growth in CEnR, see Figure 2 (and see the sidebar titled Keyword Search Strategy for complementary commentary), which is a graphical representation of references extracted from Google Scholar pertinent to different subfields of CEnR. While the extant literature suggests that CEnR is associated with greater health equity (64, 128), the underlying processes driving the effectiveness of CEnR deserve greater clarity. Assessing similarities and divergences regarding terminologies for participatory research can help inform future policy and interventions aimed at utilizing CEnR for eliminating health inequities. Our ultimate goal is to comprehensively evaluate advances across CEnR subgroups that improve effectiveness and to evaluate to what extent the published literature maps to the empirically derived CBPR model. Such an assessment can provide invaluable information for future research to strengthen dissemination and implementation for interventions utilizing CEnR.

## METHODS

### Data Sources and Search Strategy

We used the Preferred Reporting Items for Systematic Reviews and Meta-Analyses (PRISMA), integrating two validated extensions: Scoping Reviews (PRISMA-ScR; 123) and Equity (PRISMA-E; 136, 137). The protocol for our review is registered with the International Prospective Register of Systematic Reviews (PROSPERO): CRD42018101942 (see https://www.crd.york.ac.uk/prospero/display_record.php?ID=CRD42018101942). We combined the reporting guidelines of the PRISMA-ScR and PRISMA-E with guidance from the nascent but growing field of advanced scoping meta-reviews (123). Advanced scoping meta-reviews facilitate greater flexibility for assessing evidence with diverse methods deployed across included studies, which has been a challenge heretofore because most of the guidance for systematized reviews has emphasized assessment of quantitative analytics (i.e., meta-analyses).

### Eligibility Criteria

Our scoping review included studies published between January 1, 2005, and December 31, 2018, as we sought to synthesize the growth of CEnR research since the seminal 2004 Agency for Healthcare Research and Quality publication (127). Inclusion criteria were as follows:

1. English-language publication;
2. use of a systematic approach to evidence acquisition, but not necessarily meeting established requirements for systematic reviews and/or meta-analyses;
3. descriptions of partnerships from primary studies; and
4. involvement of at least one academic or research partnership assessed in the review.

Studies were excluded if they

1. did not explicitly describe the partnership or engagement with research population;
2. focused primarily on describing partnership dynamics between providers and patients, without attention to community partners;
3. did not describe at least one academic partner; or
4. were theses or dissertations.

Lastly, we did not include the gray literature, given the large number of included studies. Although a key objective was to evaluate the extent to which systematized reviews mapped onto the CBPR model, we did not exclude studies if they did not assess concepts integral to the model. Thus, our assessment of reviews permitted evaluation of construct, external, and face validity of the CBPR model while allowing possible extensions to newer iterations based on divergent concepts.

### Information Sources

A library technology informationist (J.N.), working with another team member (K.O.), searched the following databases (2005–2018): MEDLINE (PubMed), Cumulative Index to Nursing and Allied Health Literature (CINAHL), PsycInfo, Web of Science, and Google Scholar. This iterative process included initial extraction and then refinement with team members to finalize the search strategies. Initial database extractions occurred between December 2017 and January 2018. Additional database extractions occurred in April 2019 to retrieve additional systematized reviews published in 2018 using the same search strategies deployed in the previous database extraction. We also completed hand searches to identify other relevant reviews, drawing from reference lists of included studies. Team members identified potential references during the data extraction phase, and these references were evaluated for inclusion by two team leads (K.O. and J.N.). Additionally, the senior principal investigator (N.W.) shared publication alerts from Google Scholar that appeared relevant, and K.O. and J.N. filtered these suggested references to assess inclusion/exclusion and performed data extraction among studies meeting inclusion/exclusion criteria. All bibliographic content from database extractions were handled by EndNote X9, including deduplication processes.

### Search Strategy and Selection of Evidence

Complete search strategies for each database are provided in Supplemental Appendix: Search Strategy. Selection of evidence was guided by an extraction guide, which was developed by K.O. and J.N. and then finalized in consultation with the larger team (see Supplemental Appendix: Extraction Tool). This extraction tool was calibrated after two rounds of initial testing, wherein two screeners reviewed five included reviews to validate each extraction prompt for usefulness and completeness.

### Data Charting and Data Items

We used an Excel database whereby columns represented each extraction prompt from the extraction tool. In deploying a team science approach, we utilized four teams of reviewers to complete extraction processes for studies meeting inclusion/exclusion criteria. Each team of reviewers divided their total universe of reviews and compiled a database for all reviews per team. To enhance consistency of reporting and augment validity of data items, K.O. and J.N. reviewed independently the results compiled by teams to verify accuracy for all included reviews. Very few items resulted in a reconciliation process as the extraction tool was exhaustive. The evidence table, provided in Supplemental Appendix: Evidence Table, provides the data items chosen from the extraction tool. These included the following data items: (*a*) type of systematized review, (*b*) time range of review, (*c*) inclusion/exclusion criteria, (*d*) settings of included reviews, (*e*) geographical coverage of included reviews, (*f*) conceptual coverage that overlapped with the CBPR framework, and (*g*) results and findings. PRISMA-ScR (123) guidelines stipulate that quality assessments are an optional feature, as the primary goal for scoping meta-reviews is to assess broad topics of concern rather than narrowly defined research questions. Furthermore, researchers have denoted that quality assessments for scoping reviews of previously published systematic reviews should be concerned most with whether included reviews were systematically conducted as the primary function and feature of quality assessment. Because our inclusion criteria directly stipulated this feature, we did not include a formalized quality assessment.

### Data Analysis and Synthesis

Data analysis proceeded in a two-stage process. The first stage involved creating tabular representations of data items extracted from the extraction tool using Stata v15. Tabular representations allowed us to evaluate empirical contours in the growth of CEnR across our study period, along with assessment of review characteristics across all included reviews. The second stage of analysis involved detailed evaluation of a randomly generated sample of studies under each domain of the CBPR model (*n* = 5 articles per domain). Four team members (K.O., L.S., J.O., N.W.) were assigned a domain from the CBPR model. Each team member then (*a*) identified concepts from the CBPR model that mapped to concepts highlighted across reviews, (*b*) provided a synthesis of key findings across reviews, (*c*) highlighted any divergences, and (*d*) prescribed new directions to strengthen research in each domain.

## RESULTS

**Figure 3:**
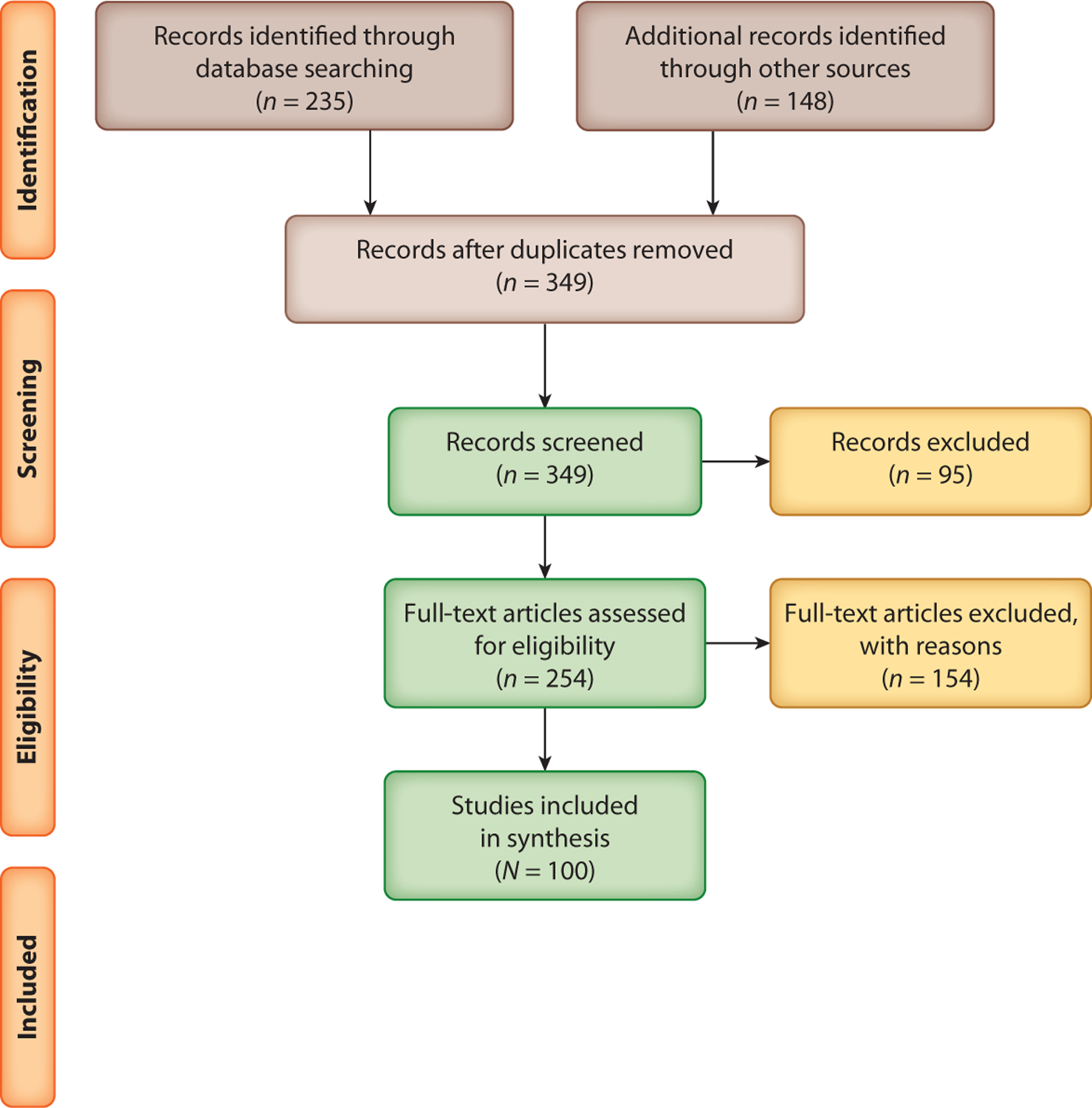
Flowchart diagram detailing the literature search.

As shown in Figure 3, initial database extractions resulted in 235 citations, which were narrowed down to *n* = 88 articles retrieved directly from databases (and *n* = 12 retrieved from additional hand searches resulting in *N* = 100 total articles) meeting inclusion/exclusion criteria after deduplication, title/abstract, and full-text review (1–5, 7–10, 12–15, 18–26, 28–33, 35, 36, 38–40, 42–54, 56, 59–61, 63, 65–70, 72, 74, 76–78, 80–87, 89–91, 97–99, 105–111, 114–122, 124–126, 135, 138–142). Through hand searches of included references, and other retrieval techniques described above, we identified an additional *n* = 148 articles after deduplication. After completing the filtering processes to ensure the studies met inclusion/exclusion criteria, we identified *N* = 100 systematized reviews included in our study population. Since 2004, there has been an increase in the diversity in types of systematized reviews used within CEnR research, with increasing sophistication as well as stronger adherence to established guidelines for systematized reviews. For example, scoping reviews did not appear to enter this literature until 2009, and since then they have exponentially grown. Studies more recently published (post 2009), with their growing adherence to reporting guidelines for systematized reviews, enhanced the possibility for study replication. Dispersed throughout the study period, basic literature reviews, anchored by systematized search processes, decreased in number, which could be driven by the growing adoption of academic journals requiring adherence to various reporting guidelines for systematized reviews. Unfortunately, our study identified only one meta-analysis that empirically evaluated, across numerous studies, elements important for successful partnering. In terms of geographic coverage, included reviews overwhelmingly emphasized CEnR studies within the United States and the Americas. Yet, a large portion of studies also derived from European countries (chiefly the United Kingdom), and a considerable number of reviews included studies that were conducted in Australia. Very few CEnR systematized reviews were found covering studies of populations in Africa and Asia.

Although we did not conduct a formal quality assessment as part of our study, we did assess the extent that included studies conducted quality assessments as part of their review process. The types of quality assessments were very diverse—so diverse that identification of groupings of quality assessments was quite challenging. Even after attempting to stratify by type of systematized review, then evaluating types of quality assessments within each of these subgroupings, we were unable to clearly identify a consistent patterning of quality assessment types. Furthermore, the depth of quality assessments varied, and most notably we identified that the quality assessments that were conducted lacked sufficient details to adequately evaluate the strength of included quality assessments. Our evaluation of included reviews also sought to understand how reviews varied in terms of whether they focused on reporting elements of CEnR relative to specific diseases or more broadly emphasized assessments of successful partnering practices. Evaluating this dimension, which we termed categorical/noncategorical, the majority of studies (56%) focused on specific diseases. However, included reviews published more recently appear to emphasize evaluating successful partnering practices more broadly, regardless of a particular disease that may have grounded partnership development. Also, types of CEnR subfields that have origins in disciplines outside of public health sciences were much less focused on specific diseases, as would be expected. Other general trends are that reviews published within the last nine years were more likely to adhere to reporting guidelines and that reviews on CEnR have been more definitively characterized by qualitative methods. We now turn attention to assessment of reviews relative to the domains of the CBPR model.

## CONTEXT DOMAIN

A total of 71 reviews (∼75%) identified concepts related to the five themes of context: social and structural, political and policy, health issue importance, capacity and readiness of stakeholders, and history of collaboration trust and mistrust. Without sufficient evaluation of the contexts in which projects are situated, collaborative efforts can be less successful. Among included reviews, social & structural contexts and health issue importance were most commonly emphasized, followed by identifying understanding of political & policy contexts, capacity & readiness, and collaboration trust & mistrust.

Stacciarini et al. (120) synthesized studies (*n* = 20) that employed CBPR to address mental health problems of racial/ethnic minorities, emphasizing strengths and challenges of CBPR within these populations. The review identified salient characteristics integral to the CBPR model—health issue importance, political and policy, and social and structural dynamics—and emphasized identifying community needs and recognizing community members as vital collaborators in research, including community gatekeepers. One critique was that traditional mental illness assessment tools and clinical instruments were still driving CBPR processes rather than having community leaders develop culturally appropriate and inclusive research approaches with minorities/underserved populations.

Coughlin & Smith’s (45) systematized literature review (*n* = 16) evaluated approaches for promoting healthy diet and nutrition and controlling obesity in African American communities, with a majority of studies highlighting social and structural dynamics and health issue importance. Coughlin & Smith concluded that CBPR approaches can be effective for African American adults, although there was limited evidence for African American youth (45), despite a bourgeoning interest in using CBPR for Latinx youth (93, 113).

McCalman and colleagues’ meta-ethnographic synthesis of PAR focused on two Australian research projects (*n* = 5), guided by empowerment frameworks and PAR methods across male advocacy groups concerned with experiences of domestic violence (87). Two context themes included reciprocal responsibility and control between academics and community partners as part of collaborative trust and mistrust. Furthermore, the sociohistorical gendered expectations of household work contributing to experiences of domestic violence stymied effectiveness of health behavior changes (87).

The scoping review by Beaulieu and colleagues (*n* = 48) proposed a conceptualization of engaged scholarship, operationalizing values, and processes (10). Two core values were identified, social justice and citizenship, which anchored community needs within an evaluation of historical social-structural barriers for successful project completion. Furthermore, boundary crossing and democratization of knowledge were stated to strengthen research processes. Beaulieu et al. highlighted a growing need for multilevel CEnR processes, as individual-level participant dynamics and institutional-level constraints (both historically and contemporaneously) can threaten the ability of academicians to conduct engaged scholarship.

Jagosh and colleagues’ (77) realist review of 276 publications, describing 23 partnerships, provided clarification in benefits and constraints of participatory research. They identified contextual factors, such as cultural histories and community capacities, but found the literature too varied to observe consistent links between context, partnering mechanisms, and outcomes. An important finding, however, was that partnerships that achieve successful outcomes can transform contexts, making their partnering more favorable for achieving future outcomes (77). This dynamic view of context is important for the CBPR model because it should not be understood as a linear model void of feedback loops across domains.

In sum, these articles support the five themes within the context domain yet more fully articulate the contextual barriers for conducting CBPR. Barriers included traditional academic practice, such as adopting validated instruments without seeking community input; challenges of reaching certain populations, such as youth; and sociocultural barriers such as gendered household expectations. Understanding these barriers, as well as recognizing facilitators of seeing contexts as dynamic, deepens the capacity of partnerships to address their contexts as part of partnering practices.

## PARTNERSHIP PROCESSES DOMAIN

A total of 73 reviews (∼77%) identified concepts within the tripartite association among individual characteristics, partnership structures, and reciprocal relationships in partnership processes. Individual characteristics include motivation(s) to participate, cultural identities, cultural humility, and reputation of principal investigator(s). Partnership structures consist of complexity and diversity of relationships among the partnership stakeholders, time in partnership, existence (or not) of formal agreements, and shared resources, which facilitate alignment with CBPR principles. Relationships are the group dynamics among partners such as participatory decision making, trust, conflict management, and dialogue. Overall, the relationship among these three domains should reflect a commitment to collective empowerment. Within this domain, the subdomain of relationships was the most commonly addressed, with some aspects of partnership structures noted. Evaluation of individual characteristics were largely absent. The most commonly relational aspects assessed were inclusion, power sharing, and shared decision making.

Anderson and colleagues’ systematic review (*n* = 58) examined community coalition-driven interventions to improve health and/or reduce health inequities in racial/ethnic minority populations, drawing on qualitative analyses to identify intervention types positively associated with behavioral and health changes (3). Although the authors recognized the diversity of partnership structures and cross-sectoral networks in building community coalitions, unfortunately they did not identify specific coalition characteristics most prominent for altering outcomes. The reviews were inconsistent in supplying sufficient evidence to generalize on these dimensions.

Bradbury-Jones and colleagues’ (13) qualitative systematic review (*n* = 13) concentrated on the methodological and practical issues in utilizing participatory research with vulnerable/marginalized children. Using thematic analytic techniques, three salient themes emerged: (*a*) importance of identifying marginalization and silenced voices, (*b*) empowerment and power (im)balances, and (*c*) dynamics of inclusion and influence. Most importantly, the authors denoted the significance of ensuring greater agency of children throughout partnering processes, as empowerment was centrally illuminated across studies in their review.

Brett and colleagues’ (19) systematic narrative review (*n* = 65) sought to understand processes involving patient and public involvement (PPI) in research in health and social care settings. Several core themes emanated: (*a*) Service users were mostly empowered via involvement, with some studies identifying participants expressing discontent as they felt disrespected or their knowledge less valued; (*b*) researchers gaining applicable insights from service users; (*c*) salience of respect and rapport-building strategies for engaging service users; and (*d*) enhancing awareness among service users relating to the severity of the health concern(s) of interest. Brett and colleagues’ (19) assessment of partnership processes indirectly described degrees of inclusion, agency (voice), and persistent community engagement strategies and denoted persistent challenges facing the prospect for health service delivery research to further integrate PPI (i.e., lack of time, money, and training).

Shamrova & Cummings’ (114) integrative methodologic review (*n* = 45) of PAR among children and youth identified three levels of PAR outcomes: outcomes for children, organizational outcomes, and community outcomes. The authors suggested that genuine participation involved trust building through training, child-friendly data collection, and involvement of children throughout the research. Although meaningful participation was not explicitly explained, it was implied that power sharing and inclusion were key.

Vaughn and colleagues’ (124) systematized literature review (*n* = 103) drew upon concept-mapping methodologies to trace impacts of community involvement for immigrant populations with complex health issues. Partnership processes focused on classifying community engagement and identifying community engagement as a continuum from low to high on the basis of amount of shared decision making, communication, and community’s level of involvement (124), reminiscent of Arnstein’s ladder of participation (6). The authors classified 61% of the articles as low to moderate engagement and the remaining as high engagement.

These five articles represent a common conceptualization of partnership processes as centered on agency, inclusion, shared power, and decision making and empowerment. These elements are key components of the CPBR model; however, exhaustive descriptions regarding individual characteristics were absent and partnership structures were evaluated minimally across the reviews, with an overreliance on implied descriptions.

## INTERVENTION AND RESEARCH PROCESSES DOMAIN

Sixty-one reviews (∼64%) reflected on how partnering processes change the development and implementation of intervention and research design, methods, and outputs. This domain includes three types of effective actions: (*a*) incorporating community and cultural knowledge into interventions/research, (*b*) empowering partners to work together well, and (*c*) involving community members throughout the research. Community involvement in all stages, from identifying health issues through disseminating and acting on results, has been identified as important for contributing to outcomes. From these actions, three types of outputs are generated: evidence of culture-centered interventions, synergy among partners to complete needed tasks, and research methods appropriate for community norms and priorities. All three processes and three outputs were noted among included reviews.

Bush and colleagues’ (25) systematic review (*n* = 107) examined the extra benefits of organizational participatory research (OPR) by extent and type of participation of health organization providers and staff within a community–academic partnership. Quantitative content analyses revealed that co-construction of research created higher benefit than consultation; benefits quadrupled when the research impetus derived from community organizations rather than from universities. With OPR, greater synergy and trust were evident in the workgroup/partnership, with four intermediate outcomes identified as highly relevant: community leadership integration, workforce development, organizational changes, and university staff transformation.

Bradford and colleagues’ (14) scoping review (*n* = 16) examined the contributions of Canadian indigenous participatory methodologies and decolonizing approaches to improve water quality. They found a lack of use of indigenous conceptions of health and water and therefore recommended greater stakeholder involvement in identifying indicators based on cultural values.

Castaneda and colleagues’ (31) critical review (*n* = 13) examined the utilization of community and organizational readiness models within health program planning. While their article is not a review of CBPR practices explicitly, they recommended greater use of these models within CBPR to better tailor interventions for communities, including attitudes of fit with community values.

Gribble & Around Him (67) identified the level of reporting on ethics and community involvement across 107 meta-analyses or systematic reviews among American Indian/Alaska Native/Native Hawaiian populations. Less than 10% reported on any approval process, i.e., seeking community input, or working with tribal institutional review boards or governments. Because only 28% of studies identified community benefits, Gribble & Around Him recommended greater attention to both ethics and participatory approaches.

Amendola’s (2) meta-synthesis (*n* = 7) assessed health care provider strategies for empowering Latinx patients. Synthesized strategies included promotores as participatory researchers, partnerships, dialogue, power sharing, and integration of culture into health care.

Similar to the majority of the 61 studies in this domain, the five studies discussed in this section show the prominence of cultural and community fit practices and involvement of community members as cocreators and also illuminate empowerment processes leading to greater synergy. They also illustrate one major element missing in the CBPR model: the importance of research ethics that privilege community benefit, beyond individual harm/benefit ratios. Studies also pointed to important nuances often not captured in reviews, i.e., the type and quality of community participation. As Bush et al. (26) note, cocreation produces more community benefit than consultation. Finally, while the other three domains focus on organizations as partners, this domain could better include organizational settings in the processes and outputs.

## OUTCOMES DOMAIN

Fifty-five included reviews (∼58%) highlighted relevant themes that can be found within the outcomes portion of the CBPR model. Although much scientific consideration has focused on the feasibility of CEnR to change health as a primary outcome, this domain is concerned with broader outcomes that are integral to CBPR principles and values. Outcomes in the CBPR model are divided into intermediate and long-term goals and include such evaluative dynamics as organizational changes in universities and their community partners, sustainability of partnerships and projects, elements of multilevel empowerment changes, changes in shared power relations in research and knowledge democracy, revitalization and cultural reinforcement, increased research productivity, enhanced financial sustainability of partnership efforts, community/social transformation, and of course greater health equity.

Chen and colleagues’ (35) systematic review (*n* = 101) sought to assess how engaged community members were involved in dissemination beyond academic publications. They found that 48% of publications identified dissemination beyond academic publication; yet among this 48%, 98% affirmed dissemination of results to community participants and 84% affirmed dissemination efforts to the general public. Soh and colleagues (117) (*n* = 21) sought to understand action research utilized within intensive care settings in the United Kingdom. They found that action research promoted effective communication as an outcome, along with greater empowerment among staff working in intensive care units (ICUs). Conversely, Soh et al. identified that action research in ICU settings also encountered challenges in identifying evaluative tools to assess feasibility and effectiveness of outcomes.

Coughlin & Smith’s (44) systematic review (*n* = 15) of CBPR methods to promote physical activity among African Americans in the United States emphasized health changes as the primary metric of an outcome. This trend was exhibited across a majority of studies wherein efforts to measure outcomes were considered. Sikorski and colleagues’ (115) scoping review (*n* = 9 randomized controlled trials) sought to understand if postnatal women’s groups improve health outcomes for mothers and children in high-income countries. In terms of outcomes that were evaluated by included studies, some of the studies evaluated single health outcomes, whereas others focused on multiple health outcomes. The health outcomes evaluated were (*a*) postnatal depression (*n* = 3), (*b*) physical activity among postnatal women (*n* = 1), (*c*) breastfeeding discontinuation, (*d*) level of fear after childbirth, (*e*) mood regulation, (*f*) life satisfaction and general well-being, (*g*) smoking, (*h*) social support, (*i*) health service utilization, and (*j*) health care–related costs. Successful intervention effects documented in included studies were identified among studies that included a psychoeducational component embedded within the intervention.

As a whole, these reviews missed several other dynamics represented in the CBPR model as possible outcomes worthy of consideration as a result of dynamic partnering practices. For example, the CBPR model emphasizes a multilevel dynamic assessment of outcomes ranging from individual-level changes in empowerment, to meso-level partnership enhancements of empowerment and power sharing, to macrolevel policy changes that can impact health for populations of interest. Disentangling outcomes within a multilevel framework can be helpful in identifying successful partnering practices that shape dynamics other than health outcomes targeted by CBPR projects. This is particularly salient considering that population-level health changes can often take considerable time, perhaps extending beyond the shelf life of specific projects. Greater attentiveness to multicomponent measured outcomes can also facilitate strengthening efforts that can shape current dissemination and implementation efforts, in particular, as investigators develop projects and fully incorporate the cyclical and iterative processes encouraged by the CBPR model.

## DISCUSSION

Evidence-based science within public health is demanding stronger evaluative tools for community and stakeholder engagement within implementation and dissemination research. Since 2004, greater emphasis within CEnR has been placed on developing empirically derived conceptual models for evaluating the effectiveness of CEnR efforts. In response, the published literature has witnessed a rapid growth of systematized reviews evaluating successful processes for CEnR, as evidenced by these 100 reviews.

As a whole, the reviews identified themes and related concepts prominently represented in the CBPR conceptual model. Although there were divergences and some additions in concepts, the four major domains held as vitally important for describing how context influences partnering processes, leading to successful community-engaged actions within research designs and interventions to achieve CBPR and health equity outcomes. Furthermore, although the CBPR conceptual model has been validated through a multimethod and multistage process, the model was never intended to be static (or necessarily linear), but instead was meant to be used as a dynamic tool to support partnerships to strengthen their collaborative processes, responses to contexts, and strategies (e.g., see http://engageforequity.org for the visioning guide for creating a partnership-specific model). Therefore, specific projects may warrant adaptations of the model and also development of empirical evaluative tools that speak directly to the unique project or discipline.

Our current study is not without limitations. The inclusion criteria stipulating only English-language publications introduced mono-language bias (27, 79, 94). We may have therefore lost the opportunity to more exhaustively capture knowledge projects developing in the Global South. Furthermore, the PRISMA framework for systematized reviews is constructed from a biomedical perspective; thus, some subfields of PAR in education or community development may not adhere to such reporting guidelines. We therefore encourage caution for interpreting the implications of our study, as considerations for using the CBPR model should be guided by principles of a specific subfield of CEnR. Additionally, selection bias is of concern because studies reporting non-null findings exhibit greater probability of publication and thus could shape the types of systematized reviews published. Another limitation of this review is that space in journal articles is limited and hence reporting was often on research design and outcomes rather than on the partnering processes themselves. The result is a “black box” where specific partnering processes that contribute to outcomes are not described sufficiently (104). For the theorizing and science of CPBR to advance (3, 100, 132), we need to identify mechanisms of partnership processes and context that contribute to key intermediate and longer-term equity outcomes.

## FUTURE DIRECTIONS

To further strengthen efforts in the development of empirically driven evaluative tools and models for CEnR, we identify several key areas that warrant future investigation and attention. First, enhanced specificity in reporting of systematized reviews could be greatly improved for CEnR. Because very few guidelines have been developed with the aim of systematically evaluating the extant literature to describe CEnR, the depth, breadth, and consistency in reporting key elements across reviews varied greatly. Relatedly, only one of the included reviews was a traditional meta-analysis, which appears to be a result of a threefold dynamic. Guidance regarding the reporting of mixed-methods designs deployed by CEnR is lacking. Many of the included systematized reviews utilized qualitative synthesis techniques, although adherence to reporting guidelines varied tremendously. Among quantitative-oriented systematized reviews, very few focused on partnering processes in describing outcomes; rather, their focus often centered on changes in health as the primary outcome. Therefore, greater attentiveness to processes shaping successful partnering dynamics is warranted and could advance the field, especially quantitative-oriented systematized reviews that deploy meta-analytic techniques. A continuing challenge facing partnerships is redressing power imbalances and positionality dynamics that may arise between community partners and academic partners. Analyses from case studies and internal team discussions have persistently revealed the significance of addressing power imbalances among partners (95, 131). Thus, empirical studies that evaluate the importance of partnerships in addressing power imbalances and positionality among partners should serve as a valuable outcome of successful partnerships.

Second, the majority of included reviews did not explicitly highlight specific concepts that were important for measurement concerns regarding effective CEnR efforts. Discrepancies between quantitative etiologies of systematized reviews within the biomedical enterprise contribute to challenges in synthesizing community engagement literature, as much of this work has been qualitative in nature. If future advances in reporting guidelines for systematized reviews included greater attention to the diversity of methodologies, while also considering the unique attributes of community engagement as a methodology and practice, researchers could accelerate construction of evaluation tools to compare more precisely the effectiveness of CEnR across its many subfields. The challenge here is that qualitative techniques can be more appropriate than quantitative measures for uncovering contexts of lived experiences and sociohistorical contributions fueling partnerships; future systematized reviews of CEnR could further incorporate mixed-methods approaches to describe contexts that contribute to partnering processes.

Third, while our focus here was to assess concepts across subfields and map them back to the CBPR conceptual model, it appears that some subfields of CEnR have progressed further in developing a measurement-focused conceptual model than have other subfields. Documenting the facilitators and the barriers to model development (i.e., limitations in structural access to resources to support funding model development) could augment dissemination and implementation science. Also, evaluating why some subfields have not sought empirical evaluative tools may provide greater insights into how such knowledge projects may not cohesively align with certain epistemic origins of particular subfields. For example, it could be that CBPR as a subfield has successfully constructed an empirically derived model as a result of its proximity to health sciences and its clinical and translational appeal, whereby investigators funded by the NIH may have been pushed for more evaluative tools that could yield quantitative reasonings. Whereas CBPR has the capacity to both bridge Western scientific knowledge production and engage with indigenous decolonizing methodologies toward goals of knowledge democracy and cognitive justice, other CEnR subfields may have less desire to accommodate Western ideals of scientific knowledge production.

Fourth, aside from tribal participatory research, which incorporates tribal governance, we found very few models, though many studies, that speak directly to specific segments of populations. For example, certain disadvantaged groups across the United States have experienced unique interactions with health care systems, thus shaping their willingness or unwillingness to participate collaboratively with health research efforts, regardless of possible shared commitment to eliminate health inequities. One such example is Drame & Irby’s 2016 (55) edited volume *Black Participatory Research*, which astonishingly claims a particular ideology for participatory research, that of an enterprise anchored by critical race theory to disrupt educational inequities rooted in structural racist educational systems. The epistemic aims of such work shares similarities with CBPR, for example in terms of privileging everyday knowledges, yet *Black Participatory Research* does not yet appear to center on a measurable model. While the public health sciences have recently seen the introduction of concepts such as critical race theory and intersectionality as important social justice frameworks, their application to much of CEnR is still marginal.

## CONCLUSION

In conclusion, the large volume of published reviews and meta-analyses on academic–community research partnerships for health demonstrates the enormous potential of this extensive and increasingly accessible CEnR approach. Many shared understandings of concepts stand out within the varied subfields of CEnR and are included within the domains of the CBPR model, including the importance of context and the principles of trust, empowerment, reciprocity, etc. These shared concepts are germane to social justice with the ultimate goal of eliminating health and social inequities.

## Supplementary Material

Evidence Table

Search Strategy

Extraction Tool

Definitions

## KEYWORD SEARCH STRATEGY

Using a Google Scholar search strategy, our team conducted a keyword search strategy for each field represented in the graphs in Figure 2 (without limiting our searches to systematized reviews, but restricting our search to studies published in the United States). This approach allowed us to understand the growth in research using these methodologies between 1994 and June 2018 [with the exception of “CBPR” and “health,” as we chose 1999 to correspond with a year following the seminal *Annual Review of Public Health* article by Israel and colleagues (75)]. In general, across all types of CEnR represented in Figure 2 graphs, one can see an exponential growth in studies deploying these methodologies. Evidence here suggests growing acceptance of these methodologies for public health research. Figure 2a indicates that action participatory research is the most widely utilized subfield of CEnR research, when compared with CBPR and PAR. Figure 2b focuses on studies using the key term “CBPR” and demonstrates a peak in 2016, with *n* = 138 studies and a steady growth of studies between 1994 and 2016. Figure 2c focuses on published research that used the key term “community-based participatory research,” and one can see year-to-year growth in the number of studies published. In comparing Figure 2b and Figure 2c, it is important to denote that studies appear to be less likely to use solely the “CBPR” acronym as a key term and are more likely to use the full spelling of “community-based participatory research.” This important distinction was also reflected in database extractions conducted within our scoping review.

## SUMMARY POINTS

1. A scoping meta-review of community-based participatory research (CBPR) and community-engaged research (CEnR) literature from 2005 to 2018 identified a rapid growth of reviews, based on an empirically derived CBPR conceptual model.
2. Developed from a 15-year research effort by the University of New Mexico with national partners, the CBPR conceptual model showcases four domains: research contexts (e.g., environments, policies, histories of collaboration), partnering processes (structural and relational dynamics among partners); intervention and research designs as outputs of shared decision making; and intermediate and long-term CBPR and health outcomes.
3. Extractions from databases resulted in 100 reviews of CEnR that largely support the CBPR conceptual framework and identify the underlying processes that drive the effectiveness of community engagement in contributing to health equity.
4. The four major domains are vitally important for describing how context influences partnering processes, leading to effective community-engaged actions within research designs and interventions to achieve CBPR and health equity outcomes.
5. Within the four domains, 76% of the reviews addressed context, 82% addressed partnership processes, 72% addressed intervention and research processes, and 67% addressed outcomes.
6. The vast majority of reviews were from the United States followed by the United Kingdom, Canada, and Australia.
7. The prominent review types were systematic (more than half), followed by scoping (∼10%). There were only three meta-analyses. The remaining most common included integrative, realist, critical, and narrative reviews (all 5% or less).

## Abbreviations

CAP: community–academic partnership
PAR: participatory action research
YPAR: youth-led participatory action research
RPP: research practice partnership
CBPR: community-based participatory research
CEnR: community-engaged research
PRISMA-ScR: Preferred Reporting Items for Systematic Reviews and Meta-Analyses—extension for Scoping Reviews
PRISMA-E: Preferred Reporting Items for Systematic Reviews and Meta-Analyses—extension for Equity
