# Supplementary material for: Partnerships, Processes, and Outcomes: A Health Equity–Focused Scoping Meta-Review of Community-Engaged Scholarship
Source: Annu Rev Public Health. Author manuscript; Available in PMC 2021 May 4. (PMC8095013; doi:10.1146/annurev-publhealth-040119-094220)
Supplement: Search Strategy [file NIHMS1551334-supplement-Search_Strategy.pdf]

Supplemental Material: Annu. Rev. Public Health 2020. 41.
https://doi.org/10.1146/annurev-publhealth-040119-094220
Partnerships, Processes, and Outcomes: A Health Equity–Focused Scoping Meta-Review of Community-Engaged Scholarship
Ortiz et al.

Supplemental Appendix: Search Strategy

PubMed: 244 Results

((((("Community Based Participatory Research" OR "Community Engaged Research" OR "Participatory Research" OR "Participatory Action Research" OR "Community Academic Partnership") OR "Community-Based Participatory Research"[Mesh])) AND systematic[sb])) OR (("Community-Based Participatory Research"[Mesh] OR "Participatory Research" OR "Participatory Action Research" OR "Community Academic Partnership" OR "Community Engaged Research")AND (((Systematic[Title] OR Scoping[Title] OR Umbrella[Title] OR Systematized[Title] OR Mapping[Title] OR Evidence[Title] OR Rapid[Title] OR "State of the art"[Title]) AND Review[Title]) OR Meta-Analysis[Title])

Web of Science: 67

# 4	100	#2 AND #3<br>*Timespan=2004-2018*<br>*Search language=Auto*	☐ ☐
# 3	194,579	#1 OR TI=((Scoping OR Umbrella OR Systematized OR Mapping OR Evidence OR Rapid OR "State of the art") AND Review)	☐ ☐
# 2	11,611	TS=("Community Based Participatory Research" OR "Community Engaged Research" OR "Participatory Research" OR "Participatory Action Research" OR "Community Academic Partnership")	☐ ☐
# 1	176,770	TI=("Systematic Review" OR "Meta-Analysis" OR (Systematic AND Literature AND Review))	☐ ☐

CINAHL (130)

#	Query	
S3	S1 AND S2	188
S2	TX "Community Based Participatory Research" OR "Community Engaged Research" OR "Participatory Research" OR "Participatory Action Research" OR "Community Academic Partnership"	6,572
S1	TI (((Systematic OR Scoping OR Umbrella OR Systematized OR Mapping OR Evidence OR Rapid OR "State of the art") AND Review) OR Meta-Analysis) OR (PT ("Systematic Review" OR "Meta Analysis"))	101,194

PsychINFO (28)

e

S6	S3 AND S4	30
S5	S3 AND S4	31
S4	TX "Community Based Participatory Research" OR "Community Engaged Research" OR "Participatory Research" OR "Participatory Action Research" OR "Community Academic Partnership"	4,749
S3	S1 OR S2	34,087
S2	MR -Systematic Review OR MR META ANALYSIS	16,861
S1	TI ((Systematic OR Scoping OR Umbrella OR Systematized OR Mapping OR Evidence OR Rapid OR "State of the art") AND Review) OR Meta-Analysis)	28,405

Search Limits: 2004 to present, only scholarly journal articles (e.g. no Dissertations or books)
