# Supplementary material for: Partnerships, Processes, and Outcomes: A Health Equity–Focused Scoping Meta-Review of Community-Engaged Scholarship
Source: Annu Rev Public Health. Author manuscript; Available in PMC 2021 May 4. (PMC8095013; doi:10.1146/annurev-publhealth-040119-094220)
Supplement: Extraction Tool [file NIHMS1551334-supplement-Extraction_Tool.pdf]

Supplemental Material: Annu. Rev. Public Health 2020. 41.
https://doi.org/10.1146/annurev-publhealth-040119-094220
Partnerships, Processes, and Outcomes: A Health Equity–Focused Scoping Meta-Review of Community-Engaged Scholarship
Ortiz et al.

SUPPLEMENTAL APPENDIX: EXTRACTION TOOL

Data Extraction Tool

General Guidelines and Tips

1. Be as specific, as brief, and as consistent in your reporting as possible. Consistency is key.
2. Some items will not be readily available in the text – don't spend too much time trying to hunt down details
3. Many reviews will have supporting/supplemental information or appendices. Sometimes relevant information on setting, participants, or other study characteristics are contained here. If you have to look here, google the article title and try to find the article on the journal website. Let Jake know if you are having trouble with this.
4. If something is **not reported**, e.g. country of origin, mark **NR**
5. If you **can't determine** information from how it is reported, mark **CD**
6. If something is **not applicable**, e.g. quality assessment in a Scoping Review, mark **NA**
7. There should be no blank cells – if something is not applicable, e.g. there are no health outcomes reported, mark NA; if you are unsure about something, mark CD and be sure to mention it in the comments.
8. Use commas to separate different items/thoughts in the same cell

NA: Not Applicable
CD: Can't Determine
NR: Not Reported

1. Citation:
   a. list 1st author last name and year in parentheses, e.g. Smith (2017)
2. Objectives:
   a. note the stated objectives of the review
   b. This should be located near the end of the introduction, or in its own section immediately following the introduction called 'Objectives'
   c. For multiple objectives, number 1) objective 1, 2) objective 2
3. Type of Review:
   a. list the type of review, e.g. Systematic Review, Scoping Review, Meta-Analysis, etc.
   b. Most reviews will identify their type in the title or when they state the objectives
4. Search Information:
   a. List all of the databases searched, e.g. PubMed, CINAHL, Google Scholar
   b. List the date range of included studies, NOT the search years, e.g. 1990-2001
   c. This should be located in the Methods section
5. Number of Studies included in final analysis and country of origin

a. List the total number of studies included in the analysis (NOT total references)
b. stratify based on location, e.g. US (n=6), MEX (n=2); if not reported, mark NR
c. This is usually located in the first paragraph of the results section, and might be located in a flow diagram, may need to look in appendix/supplemental information

6. Eligibility Criteria: Inclusion and Exclusion Criteria
   a. List each inclusion criteria identified in the review, e.g. 1) English, 2)RCT
   b. List each exclusion criteria identified in the review, e.g. 1) Review, 2) non-US publication
   c. Generally located in the Methods section, but may turn up in Results in rare occasions
7. Quality Assessment tool/process used
   a. List any tool identified in the review used to assess quality
   b. Alternatively, list the process defined in the review in lieu of a specific tool
   c. This is usually located in the Methods Section
8. Participant Details
   a. List characteristics of the individual participants/pops if described
      i. age, gender, race/ethnicity, total number summed from primary studies
   b. Generally located in Methods, or any descriptive tables or figures presented
   c. NA if not a study of specific pop, CD if you can't' determine, and NR if not reported
9. Partnership Characteristics
   a. List characteristics of partnerships if described
   b. e.g. type of partnership, number of partnerships, academic and community partners
   c. e.g. CBPR=6, CAP=4, academic departments=54, NGOs=23, community orgs=16, etc.
10. Setting
    a. refer to geographical location, but might also specify rural/urban community, clinical care setting, community based health centers, etc
    b. In Methods, or may be presented in Results or any descriptive tables or figures.
11. Context
    a. Context will either be categorical, e.g. the review is of studies on a specific health or social factor, or non-categorical, e.g. the review investigates partnership processes
    b. Record either categorical or non-categorical and the subject of investigation
    c. categorical, HIV+ community; or Noncategorical, Community engaged research common terminology and conceptual definitions
    d. In Methods, or may be presented in Results or any descriptive tables or figures.

12. <u>Findings</u>
    a. Findings from the review related to stated objectives, order and number the same as the objectives
    b. Generally located in Results and Discussion sections

13. <u>Health Related Outcomes</u>
    a. If health-related outcomes are reported, list them here
    b. Not all Reviews will report health-related outcomes, in this case mark NA

14. <u>CBPR Model Mapping</u>
    a. Using the CBPR Model and Definitions document, identify which, if any, of the CBPR model domains are expressed in the review.
    b. E.g. a review may contain aspects of both Context and Partnership Processes
    c. Write yes if present, no if absent
    d. For CBPR Model: Outcomes, mark yes or no in each of the three columns, depending on which outcome the article contains
15. <u>Other Reviews</u>
    a. While doing data extraction, if you notice reference to other reviews, copy and paste the citations in this column
    b. If you paste other reviews in this column, please also share w/ Kasim and Jake
16. <u>Comments</u>
    a. Any notes you feel necessary to leave, or if you feel unsure about how you extracted a certain item, note in this cell
    b. e.g. "I marked CD on Quality assessment because they did not describe the tool or their process, just that they did QA, pg. 8"
