# Supplementary material for: Partnerships, Processes, and Outcomes: A Health Equity–Focused Scoping Meta-Review of Community-Engaged Scholarship
Source: Annu Rev Public Health. Author manuscript; Available in PMC 2021 May 4. (PMC8095013; doi:10.1146/annurev-publhealth-040119-094220)
Supplement: Definitions [file NIHMS1551334-supplement-Definitions.pdf]

Supplemental Definitions

1. CBPR: Community-Based Participatory Research

"Collaborative efforts to gather data, build on community strengths, and co-develop multi-level strategies to improve health and social equity." (condensed from Wallerstein, et al., 2018, p. 3).

"Collaborative approach to research that equitably involves all partners, begins with research topic of importance to the community, and has action goals to eliminate health disparities." (condensed from W.K. Kellogg, 2001, in Wallerstein et al, 2018, p. 3)

Wallerstein, N., Duran, B., Oetzel, J., and Minkler, M., (eds) Community-Based Participatory Research for Health: Advancing Social and Health Equity, 3rd edition, San Francisco, Jossey-Bass, 2018.

2. CEnR: Community-Engaged Research

"The process of working collaboratively with groups of people affiliated by geographic proximity, special interests, or similar situations to address issues affecting their well-being." (CDC, 2011, p.3)

CDC, Clinical and Translational Science Awards (CTSA) Consortium, Community Engagement Key Function Committee Task Force. (2011) Principles of Community Engagement, 2nd edition. NIH, CDC, ATSDR

3. PAR: Participatory Action Research

"Orientation to research that places researcher as co-learner with community participation and translation of research findings into action for education and change" (condensed from Minkler, 2000, p. 192).

Minkler, M. (2000). Using Participatory Action Research to Build Healthy Communities. Public Health Reports 115: 191-197.

4. YPAR: Youth-Led Participatory Action Research

"Inquiry and social change that engages young people in identifying problems relevant to their lives, conducting research to understand problems, and advocating for change." (condensed from Ozer and Akemi Piatt, 2018, p.xx)

Ozer, Emily and Akemi Piatt, Amber. (201). Youth-Led Participatory Action Research (YPAR): Principles Applied to U.S. and Diverse Global Settings. In (Wallerstein, N., Duran, B., Oetzel, J., Minkler, M., Eds.) Community-Based Participatory Research for Health: Advancing Social and Health Equity. (3rd ed., pp. xx-xx). San Francisco: John Wiley & Sons.

5. PRISMA-E: Preferred Reporting Items for Systematic Review and Meta-Analysis – extension for Equity

PRISMA is an evidence-based minimum set of items for reporting, and the PRISMA-E is extending the process for equity-focused reviews.

6. PRISMA-ScR: Preferred Reporting Items for Systematic Review and Meta-Analysis – extension for Scoping Reviews

PRISMA-ScR provides readers increased knowledge of relevant terminology, core concepts, and key items to report for scoping reviews (Tricco, et al., 2018).

Tricco AC, Lillie E, Zarin W, O'Brien KK, Colquhoun H, Levac D, et al. PRISMA Extension for Scoping Reviews (PRISMA-ScR): Checklist and Explanation. Ann Intern Med. 2018;169:467–473. doi: 10.7326/M18-0850

7. Equity:

The absence of systematic disparities or major social determinants of health between social groups who have different levels of underlying social advantage/disadvantage (Braveman and Gruskin, 2003, p. 254).

Braveman P., Gruskin S. (2003) Defining equity in health. *Journal of Epidemiology & Community Health* 57:254-258.

"Health equity means that everyone has a fair and just opportunity to be as healthy as possible. This requires removing obstacles to health such as poverty, discrimination, and their consequences, including powerlessness and lack of access to good jobs with fair pay, quality education and house, safe environments, and health care. For purposes of measurement, health equity means reducing and ultimately eliminating disparities in health and is determinants that adversely affect excluded or marginalized groups."

Braveman, P. (2017). A New Definition of Health Equity to Guide Future Efforts and Measure Progress. *Health Affairs Blog*, June 22, 2017. DOI: 10.1377/hblog20170622.060710

8. RPP: Research Practice Partnerships

Long-term, mutually beneficial collaborations between education practitioners and researchers that promote the production and use of rigorous research for improved outcomes (Coburn, et al., 2013).

Coburn, C.E., Penuel, W.R., & Geil, K.E. (January 2013). *Research-Practice Partnerships: A Strategy for Leveraging Research for Educational Improvement in School Districts.* William T. Grant Foundation, New York, NY.

9. CAP: Community Academic Partnerships

“Characterized by equitable control, a cause primarily relevant to the community of interest, and specific aims to achieve a goal(s), and involves community members as well as academic researchers.” (condensed from Drahota, et al., p. 192)

Drahota, A., Meza, R. D., Brikho, B., Naaf, M. , Estabillo, J. A., Gomez, E. D., Vejnoska, S. F., Dufek, S. , Stahmer, A. C. And Aarons, G. A. (2016), *Community‐Academic Partnerships: A Systematic Review of the State of the Literature and Recommendations for Future Research*. The Milbank Quarterly, 94: 163-214. doi:10.1111/1468-0009.12184

10. Citizen Science

Inclusion of organized community knowledge to democratize and improve scientific inquiry and environmental health decision making. (Jason Coburn, Street Science, p. 3)

Coburn, J. (2005). Street Science: Community Knowledge and Environmental Health Justice. MIT Press, Cambridge, MA.
